# LINCS Dataset-Based Repositioning of Dutasteride as an Anti-Neuroinflammation Agent

**DOI:** 10.3390/brainsci11111411

**Published:** 2021-10-26

**Authors:** Dan Luo, Lu Han, Shengqiao Gao, Zhiyong Xiao, Qingru Zhou, Xiaorui Cheng, Yongxiang Zhang, Wenxia Zhou

**Affiliations:** 1 Beijing Institute of Pharmacology and Toxicology, Beijing 100850, China; 18236589692@163.com (D.L.); hanlu1@bmi.ac.cn (L.H.); gaosq_junke@163.com (S.G.); zy-xiao@163.com (Z.X.); 18260095198@163.com (Q.Z.); cxr916@163.com (X.C.); 2 State Key Laboratory of Toxicology and Medical Countermeasures, Beijing 100850, China

**Keywords:** neuroinflammation, cognitive impairment, drug repurposing, LINCS, dutasteride

## Abstract

Neuroinflammation is often accompanied by central nervous system (CNS) injury seen in various CNS diseases, with no specific treatment. Drug repurposing is a strategy of finding new uses for approved or investigational drugs, and can be enabled by the Library of Integrated Network-based Cellular Signatures (LINCS), a large drug perturbation database. In this study, the signatures of Lipopolysaccharide (LPS) were compared with the signatures of compounds contained in the LINCS dataset. To the top 100 compounds obtained, the Quantitative Structure-Activity Relationship (QSAR)-based tool admetSAR was used to identify the top 10 candidate compounds with relatively high blood–brain barrier (BBB) penetration. Furthermore, the seventh-ranked compound, dutasteride, a 5-α-reductase inhibitor, was selected for in vitro and in vivo validation of its anti-neuroinflammation activity. The results showed that dutasteride significantly reduced the levels of IL-6 and TNF-α in the supernatants of LPS-stimulated BV2 cells, and decreased the levels of IL-6 in the hippocampus and plasma, and the number of activated microglia in the brain of LPS administration mice. Furthermore, dutasteride also attenuated the cognitive impairment caused by LPS stimulation in mice. Taken together, this study demonstrates that the LINCS dataset-based drug repurposing strategy is an effective approach, and the predicted candidate, dutasteride, has the potential to ameliorate LPS-induced neuroinflammation and cognitive impairment.

## 1. Introduction

Neuroinflammation is a physiological protective response in the context of infection and injury [1], but is also an important factor contributing to cognitive impairment and neurodegenerative diseases when exceeding critical thresholds [2,3,4]. Lipopolysaccharide (LPS), a toll-like receptor ligand, commonly induces neuroinflammation [5,6]. Furthermore, a large number of studies have shown that both central and peripheral injection of LPS can induce neuroinflammation and cognitive impairment [6,7,8,9]. After intraperitoneal injection, lipid A of LPS binding to myeloid differentiation 2 (MD2) leads to recruitment of myeloid differentiation primary response protein 88 (MyD88) or toll/interleukin-1 receptor-domain-containing adaptor-inducing interferon-β (TRIF) [10,11]. The downstream signal transduction of this receptor complex activates the TLR4/nuclear factor nuclear factor-κB (NF-κB) pathway, which induces the secretion of inflammatory cytokines, such as TNF-α and IL-6, that, in turn, activate microglia [12,13] and finally cause neuroinflammation and neurodegeneration [8].

At present, the most commonly used anti-inflammatory drugs in clinics are non-steroidal anti-inflammatory drugs (NSAIDs), which have been identified as potential options for the treatment of neuroinflammation [14]. However, studies have shown that although certain NSAIDs may decrease the risk of an asymptomatic individual developing neurodegenerative disease, they result in no significant improvement in patients with basic CNS diseases, and may even worsen the diseases [15]. In addition, many other molecules have also been reported to attenuate neuroinflammation, but most of these are far from being ready for clinical use. Therefore, it is necessary to identify a new therapy for neuroinflammation from the FDA-approved drugs.

Drug repurposing is a way of identifying new uses for approved drugs, which has many virtues such as lower risks and costs, and a shorter development period [16,17]. Signature matching based on the comparison of the “signature” (characteristic) of a drug with that of other diseases or drugs [16] is an effective approach in drug repurposing. The Library of Integrated Network-based Cellular Signatures (LINCS) [18] catalogs how human cells globally respond to chemicals, and contains the largest signature dataset, including transcriptome, imaging, and proteomic data. In particular, transcriptome data in LINCS L1000 comprises the largest dataset, having more than one million signatures of 33,608 molecules [19], and is a powerful resource for drug repurposing used by numerous studies [20]. Based on a signature matching strategy, Han et al. [21] recently identified CGP-60474 as a highly potent anti-sepsis agent. Because BBB penetration is also an important factor for CNS disease treatment, we combined drug prediction based on the LINCS dataset and BBB penetration screening to develop an effective strategy to search for compounds to improve neuroinflammation and cognitive impairment.

In this study, we first obtained the top 100 compounds through signature matching, and then used the QSAR-based tool admetSAR to identify the top 10 candidate compounds with relatively high BBB penetration. Further, the seventh-ranked compound, dutasteride, a 5-α-reductase inhibitor, was selected for in vitro and in vivo validation of its anti-neuroinflammation activity. Finally, dutasteride was found to alleviate LPS-induced inflammatory cytokines secretion, microglia activation, and cognitive impairment, suggesting it has the therapeutical potential in neuroinflammation-related diseases.

## 2. Materials and Methods

### 2.1. Signature Matching and BBB Penetration Prediction

Two LPS signatures (GSE3140), generated from adult mononuclear cells and neonatal mononuclear cells (CREEDS ID: drug: 3594 and drug: 3595), were collected from CREEDS [22], which is a crowdsourcing project to annotate and reanalyze a large number of gene expression profiles from the Gene Expression Omnibus (GEO) [23]. The LINCS signatures were computed using the characteristic direction [24,25] approach using the level4 dataset, and the differentially expressed genes (DEGs) were extracted by the z-test by setting the threshold *p* values < 0.01 [26]. The pathway enrichment analysis was implemented by GSEApy [27,28].

The up-regulated DEGs of each LPS were compared with the DEGs of the 80,447 signatures of 1451 approved drugs by measuring the respective Jaccard score, and an average score was then obtained. Because the condition to generate LPS signatures was different from those of LINCS drugs, we only kept the highest Jaccard score of signatures for each drug regardless of the cell lines, drug dosage, and time point. The Jaccard score metric is shown as below, where *Si^LPS-up^* is LPS up-regulated DEGs and *S_j_^dn^* is the drug down-regulated DEGs.

$$J\left(S_{i}^{LPS\_up},S_{j}^{dn}\right)=\frac{\left|S_{i}^{LPS\_up}\cap^{\;}S_{j}^{dn}\right|}{\left|S_{i}^{LPS\_up}\cup^{\;}S_{j}^{dn}\right|}$$

Then the BBB penetration ability of the compounds with top 100 Jaccard scores was predicted by admetSAR [29,30], an QSAR-based ADMET properties prediction tool from a medicinal chemistry perspective. The BBB probability score was in the range of [–1, 1], wherein drugs with higher BBB probability were considered to be more likely to cross the BBB according to a previous study [31].

These original data and code are available at: https://github.com/gaoshengqiao/Signature-matching-based-drug-repurposing, accessed on 12 October 2021.

### 2.2. Cell Culture and Drug Administration

The immortalized BV-2 microglial cells (the Institute of Basic Medicine Chinese Academy of Medical Sciences, Beijing, China) were maintained in high glucose DMEM (Sigma, Saint Louis, MO, USA, Catalog No. D6429) supplemented with 10% FBS (Sigma, Saint Louis, MO, USA, Catalog No.12103C) at 37 °C in a humidified atmosphere of 5% CO_2_ in air.

Cells were seeded in 96 well plates at 1 × 10^5^ cells per well, then cultured for 24 h. These cells’ group distribution and administration were divided into the following groups (n = 3 per group): control group; LPS (5 μg/mL) group; LPS + TAK-242 (1 μM) group (TAK-242, a TLR4 inhibitor which can prevent LPS-induce inflammatory response, was used as positive control [32,33]); and LPS + dutasteride (10 nM–20 μM) group. Cells were incubated with TAK-242 (TargetMol, Washington, USA, Catalog No. TQ0181) and dutasteride (TargetMol, Washington, DC, USA, Catalog No. T1499) for 1 h, and subsequently stimulated with LPS (TargetMol, Washington, DC, USA, Catalog No. T11855) for 24 h, then stored for later tests.

### 2.3. Cell Viability

After LPS stimulation for 24 h, culture medium was removed and 100 μL fresh culture medium with 10 μL CCK-8 reagent (TargetMol, Washington, DC, USA, Catalog No. C0005) was added to each well. The cells continued to be cultured for 2 h. Then the cell viability was determined by measuring the optical density (OD) absorbance at the wavelength of 450 nm.

### 2.4. ELISA Measurement of TNF-α and IL-6

After LPS stimulation for 24 h, the concentrations of TNF-α and IL-6 in culture medium were detected by ELISA kit (R&D Systems, Minneapolis, MN, USA) according to the protocol recommended by the manufacturer. Absorbance was determined at 450 nm, and the concentrations were calculated according to standard curves.

### 2.5. Animals and Drug Administration

Male C57BL/6J mice (20 ± 2 g, 3 months) were purchased from Beijing SPF Biotechnology Company (license number: SCXK (Beijing) 2016–0002). During housing, the animals were kept in a temperature- and humidity-controlled room with free access to pellet food and water on a 12 h light/dark cycle. After seven days of adaptive breeding, the weight of all mice was measured. Forty-eight mice were randomly divided into four groups (n = 12 per group) according to their spontaneous motor activity: control group (equal volumes of corresponding vehicle); LPS (250 μg/kg) group; LPS + TAK-242 (3 mg/kg) group (as positive control); and LPS + dutasteride (0.65 mg/kg) group. LPS and vehicle were administered via intraperitoneal injection to induce-neuroinflammation, dutasteride was administered intragastrically, and TAK-242 was intraperitoneally injected 30 min before LPS injection. The vehicle of TAK-242 and dutasteride was a mixture (30% PEG300 + 1% Tween80 + 69% deionized water), whereas that of LPS was saline. All doses were converted according to the equivalent dose of 0.0026 for humans and mice with reference to clinical guidelines or previous studies. All dosing volumes were 0.1 mL/10 g.

### 2.6. Tissue and Blood Samples Collection

After LPS injection for 15 h, mice were collected for plasma, left hippocampus, and right hemisphere samples. The tissues were quickly collected and then placed in liquid nitrogen for quick freezing, and then placed in a −80 °C refrigerator for later tests.

### 2.7. MWM Test

The MWM test was consistent with the method of Wang et al. [34], including hidden-platform training (spatial learning) and probe trial (spatial memory) sessions. In the hidden-platform training session, mice were allowed 4 daily trials in the presence of the platform, for 4 or 5 subsequent days. In the probe trial session (on the fifth or sixth day of the experiment), the platform was removed, and mice were allowed to swim to search it for 60 s. The escape latency (the time taken to find the hidden platform) in the hidden-platform training sessions and the escape latency (the first time that the mice crossed the removed platform), time in the target quadrant, and number of crossings of the removed platform in the probe trial sessions were recorded and analyzed. To develop acute neuroinflammation, mice were pretreated with dutasteride or TAK-242 for 30 min, then treated with LPS after the first day of the MWM test, and subsequently continued to undergo tests. To develop chronic neuroinflammation, mice were pretreated with dutasteride or TAK-242 for 30 min before LPS treatment daily for two months, then continued to undergo MWM tests

### 2.8. Immunofluorescence

According to the experimental protocol of Campos et al. [35], five micrometer thick paraffin sections were prepared from the hemispheres of 5 mice. After dewaxing, blocking and other operations, these paraffin sections then were incubated with Iba1 antibody (Abcam, Cambridge, UK, Catalog No. ab5076) (diluted 1:1000 in PBS) overnight at 4 °C. Washing with PBS was followed by incubation with IgG (H + L)/HRP antibody (Zhong Shan Jin Qiao Corporation, Beijing, China, Catalog No. ZB-2306) (diluted 1:1000 in PBS) for 30 min at room temperature. Immunofluorescence images were acquired using fluorescence microscope (Leica Microsystems Inc, Wetzlar, Germany). Finally, cell integrated optical density (IOD) of IBA-1 was detected in the hemispheres of mice using Image Pro Plus 6.0 software.

### 2.9. Luminex Assay Detection of Multiple Cytokines

The concentrations of IL-6, IL-1β, TNF-α, RANTES, GM-CSF, MCP-1, IFN-γ, IL-4, IL-10, and VEGF in plasma and hippocampal homogenate were determined by a Luminex kit (Merck, Darmstadt, Germany, Catalog No. 3100931). Briefly, 10 μL/well of antibody-immobilized beads were co-incubated with 10 μL/well of diluted sample for 60 min, then carefully washed 3 times with 200 μL of wash buffer per well, followed by incubation with 10 μL of detection antibody and streptavidin-phycoerythrin per well for 30 min. After the final washing step, 150 μL of assay buffer was added to each well, and the plates were analyzed using the Luminex 200 (Luminex Crop, Austin, TX, USA) according to the manufacturer’s protocol.

### 2.10. Statistical Analysis

Data are expressed as mean values ± standard deviation. The statistical analyses were performed with GraphPad Prism 8.0 software. One-way ANOVA followed by Dunnett’s test or one-way ANOVA followed by Tukey’s test were used in assessing comparisons between groups, and *p* < 0.05 was considered to be statistically significant.

## 3. Results

### 3.1. Repurposing of Dutasteride to Treat Neuroinflammation by Signature Matching and BBB Penetration Screening

**Figure 1:**
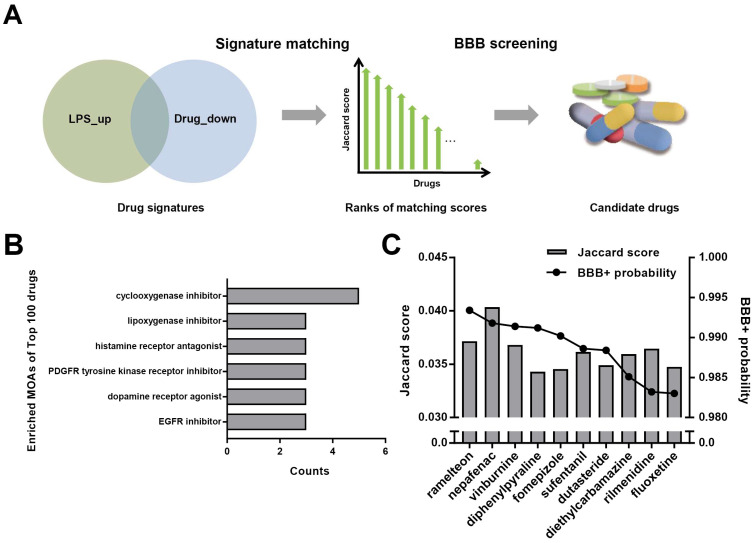
Candidate drug identification and evaluation. (**A**) The workflow of candidate drug identification. (**B**) The most enriched mechanism of actions among the top 100 drugs. (**C**) Ten drugs with the highest BBB+ probability among the top 100 drugs and corresponding Jaccard scores.

The workflow of drug screening is shown in Figure 1A. Firstly, we analyzed the enriched pathway of LPS signatures (CREEDS ID: drug: 3594 and drug: 3595), in which the LPS up-regulated genes were significantly enriched in innate immunity pathways, such as TNF and NF-κB pathways (Figure S1A), consistent with its clinical symptoms. We hypothesized that small molecules that down-regulated this gene expression state may decrease the inflammatory responses. Therefore, the LPS up-regulated genes and drug down-regulated genes were compared in this study as illustrated in Figure 1A. Jaccard similarity was utilized between signatures of 1451 approved drugs and those of LPS to search for the leading compounds that oppose LPS. Then we analyzed the mechanism of actions (MOAs) of the top 100 compounds. The most enriched entities (Figure 1B) included cyclooxygenase inhibitors and lipoxygenase inhibitors, which were reported to be able to alleviate the LPS responses, implying the signature matching approach would be reliable. The top 100 compounds were subsequently screened by admetSAR [29,30] to obtain a BBB probability score. The BBB probability score was in the range of [−1, 1]. According to previous study, molecules with higher BBB probability were considered to be more likely to cross the BBB [31]. Here, we retained 10 molecules with the highest BBB probability (Figure 1C).

Further document retrieval showed that the 10 candidate compounds, with the exception of dutasteride and fomepizole, were all revealed to possess anti-inflammatory or neuroprotective effects in a large number of studies. This evidence also indicates that our strategy is of high accuracy. We focused on dutasteride particularly, and further analyzed the reversal overlapped genes between dutasteride and LPS. The overlapped DEGs of dutasteride and LPS (*p* value = 1.01 × 10^−17^, hypergeometric test), were significantly enriched in TNF and NF-κB pathways (−log adjust *p* value > 3, Figure S1B), suggesting dutasteride and LPS may modulate innate immunity pathways in reverse directions. Therefore, we speculated dutasteride may play a role in LPS-induced neuroinflammation.

### 3.2. Dutasteride Reduced Cytokine Secretions in LPS-Activated BV2 Microglial Cells

**Figure 2:**
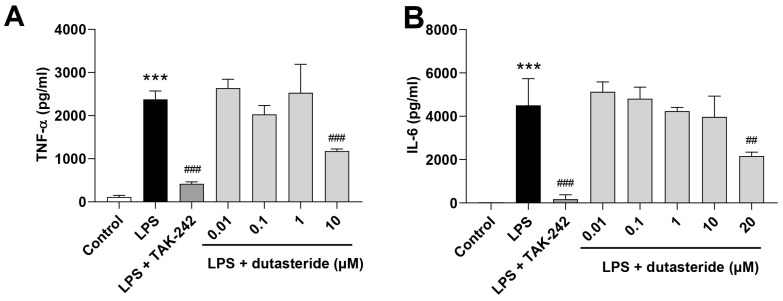
Dutasteride reduced the concentrations of proinflammatory cytokines in LPS-activated BV2 microglial cells. (**A**) The level of TNF-α secretion. (**B**) The level of IL6 secretion. Above data are presented as the mean ± S.D (*n* = 3 per group); *** *p* < 0.001 compared with the control group (Control); ^##^ *p* < 0.01, ^###^ *p* < 0.001 compared with the LPS group (Model).

Microglia is an active participant in neuroinflammation by releasing inflammatory cytokines [8,36].The immortalized murine BV-2 microglia cell line is widely used in experimental neuroinflammatory studies [37,38]. To elucidate the anti-neuroinflammatory efficacy of dutasteride, LPS (250 μg/mL) was used to activate BV2 microglial cells, and TAK-242 (1 μM), a TLR4 inhibitor which can prevent LPS-induce inflammatory response [32,33], was used as positive control. We firstly investigated the concentration-dependent effects of dutasteride (0.01, 0.1, 1, 10, 30, 50 μM) on the survival of BV2 microglial cells (Figure S2A). Then at non-cytotoxic concentrations (0.01, 0.1, 1, 10, 20 μM) (Figure S2B), the levels of IL-6 and TNF-α secreted in LPS-stimulated BV2 microglial cells with LPS treatment for 24 h were measured by ELISA. The results showed that dutasteride pretreatment significantly reduced the level of TNF-α and IL-6 induced by LPS (TNF-α: *p* < 0.001, IL-6: *p* < 0.01; Figure 2A,B), indicating that dutasteride can alleviate inflammatory response at the cellular level.

### 3.3. Dutasteride Decreased Level of IL-6 in Plasma and Hippocampus following LPS Administration

**Figure 3:**
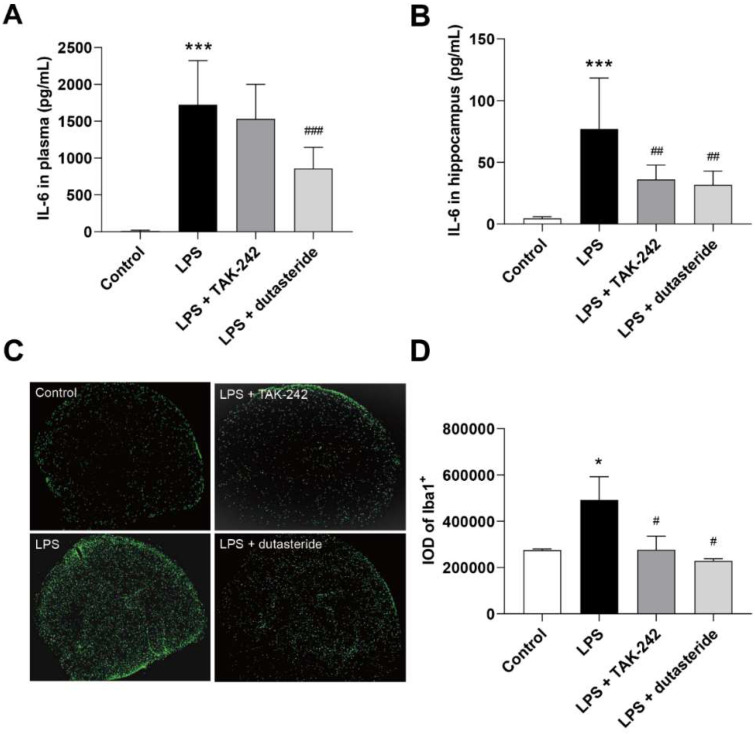
Dutasteride suppressed inflammatory reaction in the LPS-induced neuroinflammatory mouse model. (**A**) The concentration of IL-6 in plasma (*n* = 8~12 per group). (**B**) The concentration of IL-6 in hippocampus (*n* = 8~12 per group). (**C**,**D**) The examination of IBA-1 immunohistochemical staining (microglia activation) of the semi-brain (*n* = 3 per group). Above data are presented as the mean ± S.D; * *p* < 0.05, *** *p* < 0.001 compared with the control group (Control); ^#^ *p* < 0.05, ^##^ *p* < 0.01, ^###^ *p* < 0.001 compared with the LPS group (Model).

To investigate the effect of dutasteride on neuroinflammation in vivo, mice were pretreated with dutasteride (0.65 mg/kg) or TAK-242 (3 mg/kg) for 30 min, then treated with 250 μg/kg LPS for 15 h. The concentrations of inflammatory cytokines in the plasma and hippocampus were measured. The results are shown in Table S1, where the levels of IL-6 in the plasma and hippocampus significantly increased after LPS injection (plasma and hippocampus: *p* < 0.001; Figure 3A,B), and dutasteride administration effectively reduced LPS-induced production of IL-6 in plasma and hippocampus (plasma: *p* < 0.001, hippocampus: *p* < 0.01; Figure 3A,B). In addition, dutasteride administration also significantly increased the ratio of thymus to body weight compared to the LPS-treated group (*p* < 0.05; Figure S3A). These results show that dutasteride can effectively attenuate LPS-induced inflammation both in the central and peripheral systems.

### 3.4. Dutasteride Decreased Microglia Activation in the Brain of LPS Stimulated Mice

LPS-induced neuroinflammation is accompanied by microglia activation. Here we further investigated the inhibitory effect of dutasteride on brain microglia activation on as outlined in Section 3.3. The results showed that LPS administration significantly increased the number of activated microglia (IBA-1 labeled) in the brain of mice (*p* < 0.05; Figure 3C,D), whereas dutasteride treatment decreased the number of activated cells compared to the LPS-treated group (*p* < 0.05; Figure 3C,D). These results show that dutasteride can effectively attenuate the activation of microglia cells caused by LPS.

### 3.5. Effects of Dutasteride on Spatial Learning and Memory in LPS-induced Neuroinflammatory Mouse Model

**Figure 4:**
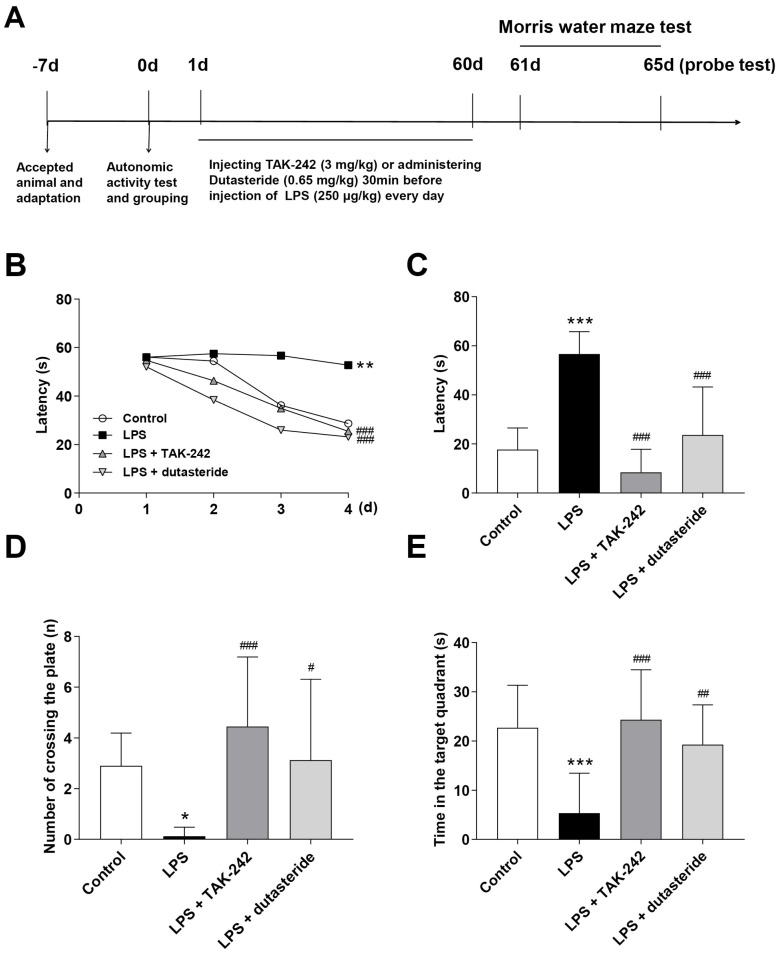
Effects of dutasteride on spatial learning and memory in LPS-induced neuroinflammatory mouse model. (**A**) Workflow: the effect of dutasteride on the learning and memory of chronic neuroinflammation caused by multiple injections of LPS. (**B**) The escape latency during the learning session of the MWM tests. (**C**) The escape latency, (**D**) the number of platform area crossings, and (**E**) the time spent in the target quadrant during the probe test of the MWM tests. Data are presented as the mean ± S.D (*n* = 10~12); * *p* < 0.05, ** *p* < 0.01, *** *p* < 0.001 compared with the control group (Control); ^#^ *p* < 0.05, ^##^ *p* < 0.01, ^###^ *p* < 0.001 compared with the LPS group (Model).

Here we designed single and long-term intraperitoneal injection of LPS to induce acute and chronic neuroinflammation in the mouse. The effects of dutasteride on cognitive impairment were detected via MWM test, a standard and routine method to assess spatial learning and memory ability in rodents [39]. The acute neuroinflammatory model (the experimental schedule is shown in Figure S4A) showed that dutasteride administration significantly decreased the escape latency compared to the LPS-treated group in the first day after LPS injection (Day 2: *p* ˂ 0.05; Figure S4B), but no significant was found in the following training and test sessions, both in the LPS-treated group and the dutasteride-treated group (Figure S4B–E), indicating that single injection of LPS (low doses, 250 μg/kg) induced cognitive impairment that was time-limited, which was consistent with previous studies [6,40]. In the chronic neuroinflammatory model (the experimental schedule is shown in Figure 4A), after multiple intraperitoneal injections of LPS for two months, the LPS-treated mice exhibited long-term learning impairment, and LPS-treated mice showed a longer escape latency than the control group on the last day of training. Dutasteride significantly decreased the escape latency compared with the LPS-treated group (Day 4: *p* ˂ 0.001; Figure 4B). Similarly, during the probe test period, dutasteride administration significantly decreased the escape latency compared with the LPS-treated group (*p* ˂ 0.001; Figure 4C). Moreover, the dutasteride-treated group showed a significant improvement in the number of platform area crossings (*p* ˂ 0.05; Figure 4D) and the time spent in the target quadrant (*p* ˂ 0.01; Figure 4E) compared with the LPS-treated group (Figure 4D,E). In summary, these results suggest that dutasteride can alleviate LPS-induced chronic or persistent neuroinflammation and cognitive impairment.

## 4. Discussion

Neuroinflammation is a key pathological event triggering and perpetuating the neurodegenerative process associated with many CNS diseases [6], and is a complex process that is related to systematic dysregulation of cellular functions or pathways [3]. It is well-known that an omics dataset can represent the systematic effect of organisms/cells exposed to chemicals. Therefore, it is an abundant source for discovering therapeutic candidates. LINCS transcriptome data constitutes the largest dataset, having more than one million signatures of 33,608 molecules [19], and has been previously demonstrated as a powerful resource for drug repurposing in numerous studies [20]. Additionally, several successful cases have also proved the reliability of the LINCS resource in discovering drugs to oppose inflammatory related disease or responses, including inflammatory bowel disease [41], LPS induced sepsis [21], and cigarette smoke-induced inflammation [42]. However, for the treatment of neuroinflammation, many drugs examined in research failed in clinical trials because they do not effectively cross the blood–brain barrier (BBB) [43,44,45,46]. Therefore, an additional screening based on BBB penetration is necessary to identify compounds of greater potential therapeutic value.

In this study, we used LINCS dataset-based signature matching followed by BBB penetration screening to search for potential anti-LPS-induced neuroinflammation agents, and found that most of the top 10 candidate compounds, with the exception of fomepizole, have been reported to have anti-inflammatory or neuroprotective effects. For example, ramelteon and fluoxetine can relieve neuroinflammation [33,47,48], and rilmenidine and vinburnine have been reported to have neuroprotective effects [49,50,51]. Moreover, nepafenac, diphenylpyraline [52,53], sufentanil [54], dutasteride [55], and diethylcarbamazine [56,57] have also been found to have anti-inflammatory effects, indicating that this strategy contributes to identifying potential compounds having anti-neuroinflammation properties among approved and marketed drugs. The lengthy time to develop new drugs and the hindrance posed by the BBB are the main obstructions for uncovering novel treatments for neuroinflammation [58]. We think there are two essential conditions needed to be considered in the discovery of anti-brain neuroinflammation drugs: the ability to inhibit the pathological processes of inflammation, and the ability to penetrate the BBB. However, it cannot be ruled out that some drugs can affect the inflammatory processes of the central nervous system through other indirect processes. The prediction strategy based on LINCS data and BBB penetration in this study is not only time- and cost-effective, but also takes into account the role of the BBB in CNS diseases, so as to improve the successful rate of drug repurposing, and may be applied in other CNS diseases.

Among the top 10 compounds examined above, most have been revealed to possess anti-inflammatory or neuroprotective effects in a large number of studies. In contrast, dutasteride has been rarely reported. Therefore, for the purpose of identifying new potential anti-neuroinflammatory molecules and our research focus, we selected dutasteride for further investigation in in vitro and in vivo experiments. The results showed that dutasteride significantly reduced the secretion of IL-6 and TNF-α in LPS-stimulated BV2 cells, and decreased the microglia activation in the brain and the levels of IL-6 in the plasma and hippocampus in an LPS-induced neuroinflammatory mouse model, indicating that dutasteride can effectively suppress the inflammatory response stimulated by LPS in the peripheral and central nervous systems. Additionally, our MWM tests results showed that dutasteride can alleviate LPS-induced learning and memory impairments. Thus far, our study demonstrates that dutasteride can ameliorate LPS-induced neuroinflammation and cognitive impairment.

The direct anti-neuroinflammatory and neuroprotective mechanisms were not further investigated in this study. According to previous research, dutasteride is a selective inhibitor of type 1 and type 2 5-α-reductase, a family of several isozymes responsible for the conversion of testosterone to 5-α-dihydrotestosterone [59], and mainly used to clinically treat benign prostatic hyperplasia (BPH). The inhibition of 5-α-reductase can increase the level of testosterone [60] and promote the conversion of testosterone to 17-β estradiol [61]. Increasing evidence suggests that both testosterone and 17-β estradiol can inhibit the activation of microglia cells, thereby playing an anti-inflammatory role [62,63]. In addition, several studies reported the neuroprotection effect of dutasteride, and proposed that the neuroprotection of 5-α-reductase inhibitor is an androgen receptor-dependent mechanism [61,64,65]. These may provide an explanation for the mechanism of dutasteride against neuroinflammation. Interestingly, however, we noted that the results in Figure 2 showed that the concentrations of TNF-a and IL-6 with low-dose (0.01, 0.1, 1 μM) dutasteride treatment were slightly higher than those with LPS treatment (no significant difference). This phenomenon may be due to the potential pro-inflammatory effect of dutasteride at low doses and anti-inflammatory effect at high doses. As reported in previous studies, long-term low-dose use of 5-α-reductase inhibitors has side effects on the central nervous system, including anxiety, depression, and suicidal ideation [66,67,68], which are highly correlated with neuroinflammation reactivation [69]. By comparison, dutasteride was reported to be able to inhibit Keap1-Nrf2 interaction, an essential pathway involved in oxidative stress [55]. Studies have shown that the inhibition of Keap1-Nrf2 interaction can eliminate ROS or inhibit the transcription of pro-inflammatory cytokine genes [70,71,72] or inhibit NF-κB activation, thereby suppressing inflammation [73]. Therefore, inhibition of keap1Keap1-nrf2 Nrf2 interaction may also partly explain the anti-neuroinflammatory mechanisms. Nonetheless, the anti-neuroinflammatory characteristics of dutasteride and its mechanism of action need to be further studied and elucidated.

## 5. Conclusions

In summary, we repurposed 1451 compounds using the LINCS dataset and BBB penetration screening-based virtual prediction. One of the top 10 compounds, dutasteride, was validated to possess anti-neuroinflammatory and cognitive improvement effects in vitro and in vivo. These results indicated that signature matching combined with BBB penetration screening is an effective method for drug prediction for CNS disease. The predicted candidate, dutasteride, may be worthy of further evaluation as an anti-neuroinflammation agent, and may provide a potential therapeutic option for neuroinflammation-related diseases.

## Data Availability

The datasets used and/or analyzed during the current study are available from the corresponding author on reasonable request.

## Supplementary Materials

The following are available online at https://www.mdpi.com/article/10.3390/brainsci11111411/s1, Supplementary Files: Table S1. Profiling of plasma and hippocampus cytokine level. Figure S1. Enrichment analysis of overlapped genes between dutasteride and LPS with KEGG pathway database. Figure S2. Effects of dutasteride on the survival rate of BV2 cells and LPS-stimulated BV2 cells. Figure S3 Effect of dutasteride on the ratio of thymus to body weight following LPS administration. Figure S4 Effects of dutasteride on spatial learning and memory in a single injection of LPS-induced neuroinflammatory mouse model.
