# Supplementary material for: LINCS Dataset-Based Repositioning of Dutasteride as an Anti-Neuroinflammation Agent
Source: Brain Sci. 2021 Oct 26;11(11):1411. doi: 10.3390/brainsci11111411 (PMC8615696; doi:10.3390/brainsci11111411)
Supplement: Supplementary file 1 [file brainsci-11-01411-s001.zip › brainsci-1401853-supplementary.pdf]

Supplementary Information

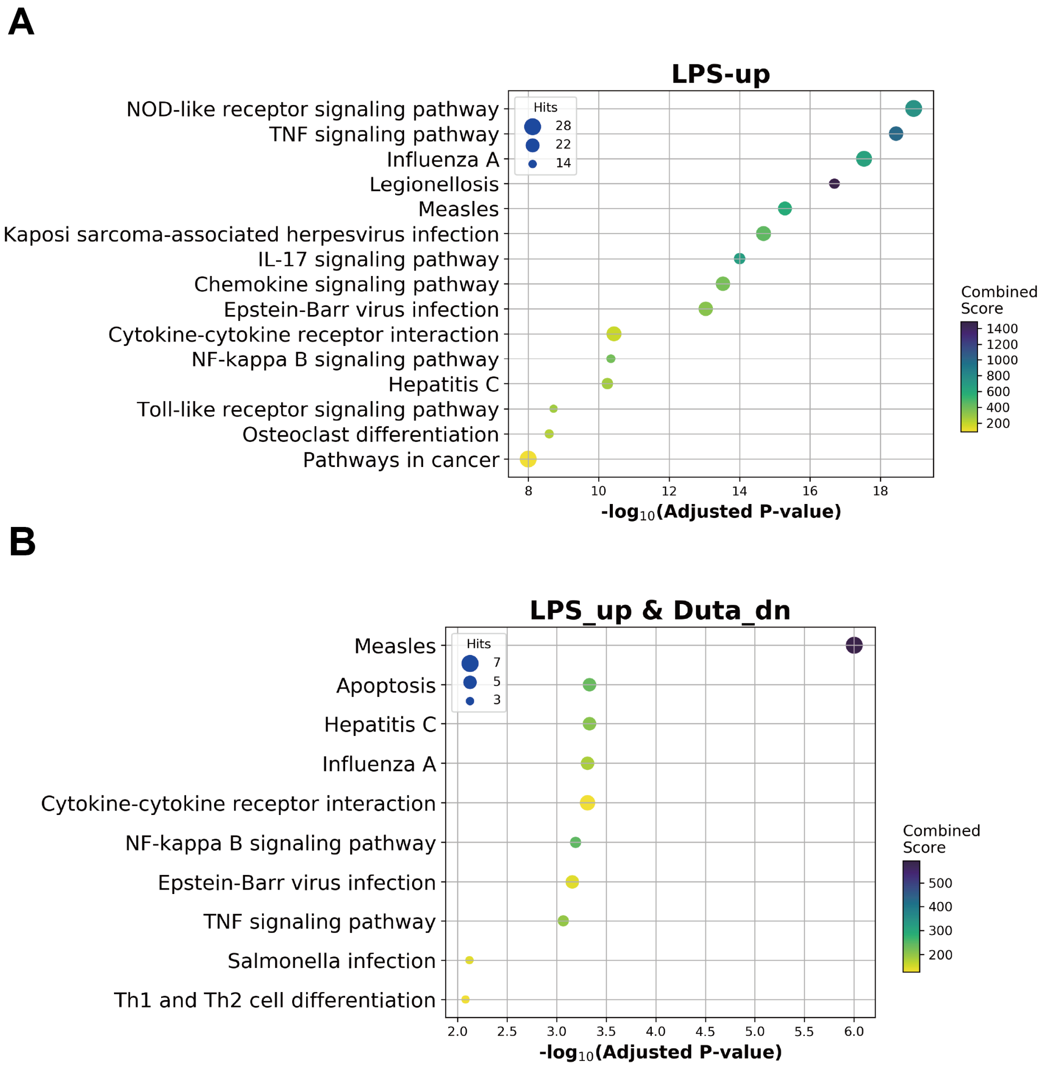

Figure S1. (A) KEGG enrichment analysis of LPS up-regulated genes, the enriched pathways includes inflammation pathways such as TNF and NF-κB pathways. (B) The overlapped genes between Dutasteride down-regulated DEGs (Duta_dn) and LPS up-regulated DEGs (LPS_up) are enriched to pathways such as TNF and NF-κB pathways which is consistent with (A) LPS enriched pathways.

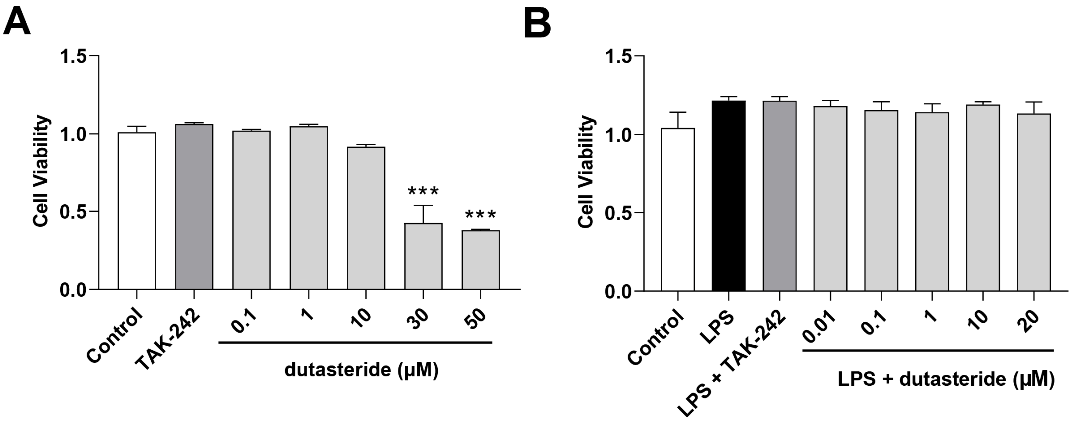

Figure S2. (A) (B) Effects of Dutasteride on the survival rate of BV2 cells and LPS-stimulated BV2 cells. Above of Data are presented as the mean ± S.D (n=3) ; ***$P<0.001$ compared with the control group (Control).

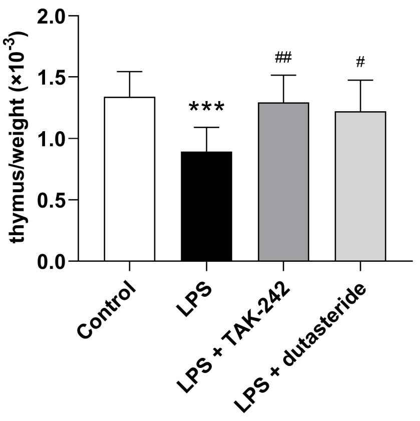

Figure S3. Effect of Dutasteride on the ratio of thymus to body weight following LPS administration. Above of Data are presented as the mean ± S.D (n=8～12 per group); $^{***}P<0.001$ compared with the control group (Control); $^{\#}P<0.05$, $^{\#\#}P<0.01$ compared with the LPS group (Model).

Table S1. The effect of Dutasteride on the level of inflammatory cytokines in plasma and hippocampus of LPS-induced neuroinflammatory mouse model.

Tissue	Group	IL-6 ng/L	IL-1β ng/L	TNF-α ng/L	RANTES ng/L	MCP-1 ng/L	GM-CSF ng/L	IL-4 ng/L	IL-10 ng/L	VEGF ng/L
Plasma	Control	11.9±11.3	N/A	6.0±4.5	23.5±18.7	N/A	N/A	N/A	4.3±2.1	N/A
	LPS	1725.0±598.8***	N/A	13.4±5.7	127.0±39.7***	N/A	N/A	N/A	167.2±62.5***	N/A
	LPS + TAK242	1533.0±467.2	N/A	12.0±8.5	82.6±16.5$^{\#}$	N/A	N/A	N/A	104.6±15.7$^{\#\#}$	N/A
	LPS + dutasteride	859.7±286.1$^{\#\#\#}$	N/A	14.3±7.1	125.4±32.4	N/A	N/A	N/A	180.2±53.6	N/A
Hippocampus	Control	4.6±1.4	41.0±24.9	6.2±5.7	6.2±5.7	148.3±102.5	36.0±30.5	0.9±0.9	142.6±57.6	3.4±1.7
	LPS	77.0±41.4***	43.4±16.9	7.3±4.3	17.5±4.5***	394.1±207.9*	41.7±24.6	0.8±0.5	114.0±33.4	3.8±1.3
	LPS + TAK242	36.0±11.7$^{\#\#}$	40.7±12.3	9.8±6.4	11.2±5.9$^{\#}$	193.1±23.1$^{\#}$	30.5±23.9	1.0±0.4	249.3±138.6$^{\#\#}$	4.7±1.1
	LPS + dutasteride	31.8±11.1$^{\#\#\#}$	33.9±15.5	5.4±3.3	14.7±5.8	295.4±229.5	16.2±12	0.6±0.3	136±36	4.0±1.9

IL: interleukin; TNF: tumor necrosis factor; RANTES: regulated upon activation normal T cell expressed and secreted; MCP: monocyte chemoattractant protein; GM-CSF: granulocyte-macrophage colony-stimulating factor; VEGF: vascular endothelial growth factor; N/A: the sample concentration is outside the range of the calibration; Above of Data are presented as the mean ± S.D (n=10～12 per group); $^{*}P<0.05$, $^{***}P<0.001$, compared with the control group (Control); $^{\#}P<0.05$, $^{\#\#}P<0.01$, compared with the LPS group (Model).

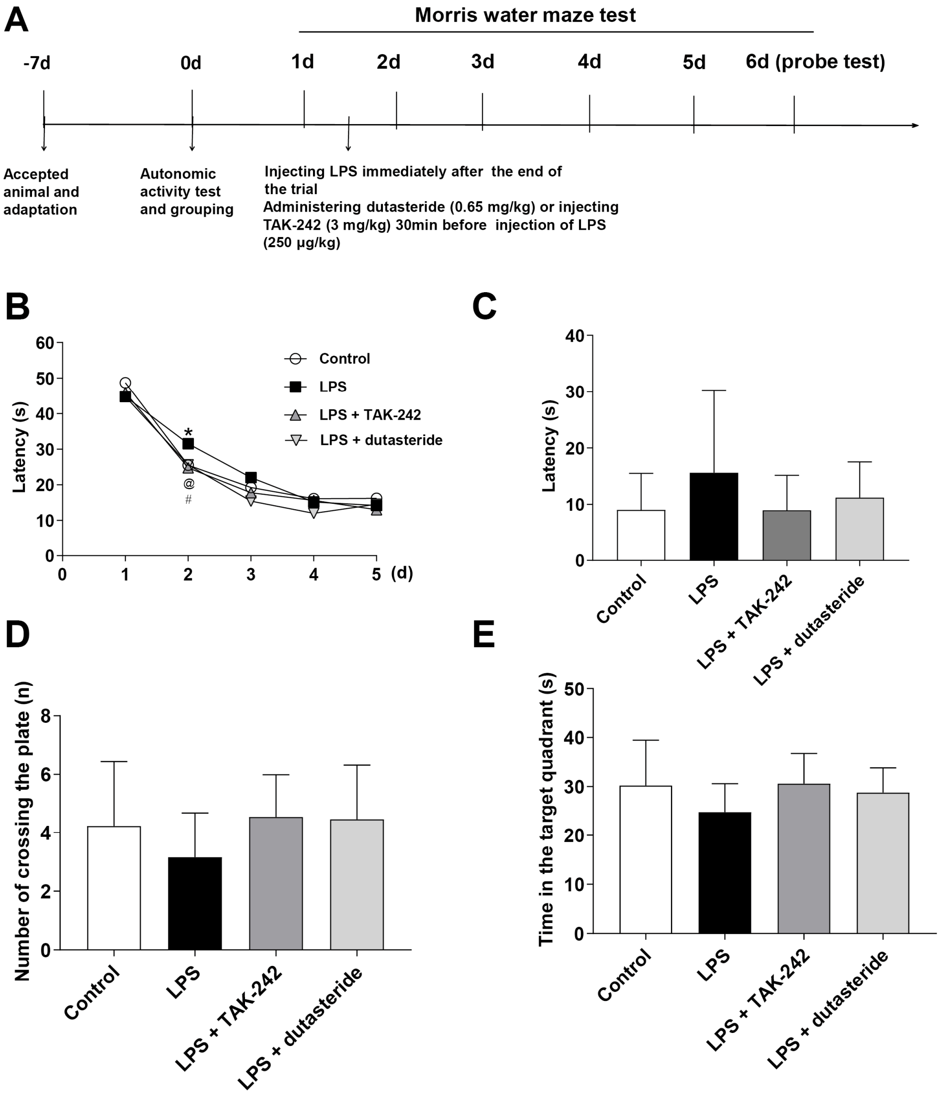

Figure S4. Effects of Dutasteride on spatial learning and memory in a single injection of LPS-induced neuroinflammatory mouse model (A) Workflow: the effect of Dutasteride on the learning and memory of acute neuroinflammation caused by single injection of LPS. (B) The escape latency during the learning session of the MWM tests. (C) The escape latency, (D) the number of platform area crossings and (E) the time spent in the target quadrant during probe test of the MWM tests. Data are presented as the mean ± S.D (n=10～12 per group); *$P<0.05$ compared with the control group (Control); #$P<0.05$ (LPS+TAK-242), @$P<0.05$ (LPS + dutasteride) compared with the LPS group (Model).
